# Monitoring Performance in Show Jumping Horses: Validity of Non-specific and Discipline-specific Field Exercise Tests for a Practicable Assessment of Aerobic Performance

**DOI:** 10.3389/fphys.2021.818381

**Published:** 2022-01-14

**Authors:** Katharina Kirsch, Christina Fercher, Stephanie Horstmann, Caroline von Reitzenstein, Julia Augustin, Henrike Lagershausen

**Affiliations:** ^1^German Olympic Committee for Equestrian Sports, Warendorf, Germany; ^2^Olympic Training Center NRW/Westphalia, Warendorf, Germany

**Keywords:** horses, show jumping, exercise test, heart rate, lactate, GPS

## Abstract

Show jumping is a highly specialized equestrian discipline that requires technical skill but also power and fitness. Monitoring the horses’ aerobic performance is therefore essential in order to verify whether the training has induced the desired cardiovascular and muscular adaptations. This study therefore aimed at evaluating the validity of non-specific and discipline-specific field exercise tests for objective evaluation of aerobic performance in show jumpers. For this purpose, data obtained from horses competing at Junior and Young Rider level during show jumping competitions as well as field exercise tests were retrospectively analyzed. The effect of the level of difficulty, the horses’ age, the penalty score and the horses’ previous level of performance on blood lactate concentrations after show jumping competitions (100 observations in 49 horses) was evaluated by linear mixed effects models (horse as random effect). Estimated marginal means significantly increased from 140 (4.1 mmol/L) to 150 cm (5.2 mmol/L) classes (*P* = 0.02). Furthermore, post-exercise lactate values significantly increased with the horses’ age (*P* = 0.001). Another group of 12 horses performed a standardized incremental field exercise test on a track (SET_track_), a standardized show jumping course (SET_course_) and a standardized grid exercise (SET_grid_) each on three consecutive days. Indices of aerobic performance, derived from the SET_track_ [velocity at a heart rate of 140 bpm (V_140_) and at a lactate concentration of 2 mmol/L (V_*La2*_)] were highly correlated with heart rate (V_140_: r = −0.75, *P* = 0.005; V_*La2*_: r =−0.66, *P* = 0.02) and lactate (V_140_: r = −0.73, *P* = 0.02; V_*La2*_: r = −0.72, *P* = 0.02) in response to SET_course_ as well as heart rate during SET_grid_ (V_140_: r = −0.73, *P* = 0.02; V_*La2*_: r = −0.76, *P* = 0.01). Subjective rating of muscular fatigue was significantly correlated to the mean heart rate during SET_course_ (r = −0.64, *P* = 0.05) and SET_grid_ (r = −0.74, *P* = 0.02) but not to the aerobic indices calculated from SET_track_. Besides non-specific incremental field tests, performance monitoring in show jumpers should therefore also include discipline-specific tests that more closely reflect the internal load induced by show jumping competitions.

## Introduction

In contrast to disciplines in which success is either primarily based on endurance (endurance horses) or speed (Thoroughbred racehorses or Standardbred trotters), the requirements for a show jumping horse are more complex in that show jumping requires a combination of speed, power, strength endurance and technical skills. Show jumpers need to be able to generate the explosive power that is required to clear demanding obstacles but at the same time are required to complete multiple courses at the same or over consecutive days which requires the ability to recover quickly between successive bouts of exercise. At Junior (U18) and Young Rider (U21) European Championship level, the horses have to complete up to three competitions (Qualification, Team Final, and Individual Final) throughout 3–4 days. The first qualifying competition consists of one round, the Team Final of two rounds over 500–600 m at speeds of 375 m/min for Juniors and 400 m/min for Young Riders with 12–14 obstacles at maximum heights of 140 cm for Juniors and 150 cm for Young Riders. The Individual Final consists of one round over 500–600 m with 10–12 obstacles and a second round over a 450–550 m with 8–10 obstacles. In the Team and the Individual Final, the horses have to complete an additional jump-off over a reduced course of 6 obstacles in case of equality of penalties from the first two rounds (FEI Jumping Rules, 2021).

It is well established that physical exercise induces transient alterations in the horses’ homeostasis that need to be restored by cardiovascular, respiratory, metabolic, and musculoskeletal adaptations (Assenza et al., 2018; Arfuso et al., 2020, 2021a). Among the parameters considered for the evaluation of athletic performance in horses, cardiovascular parameters are the most studied as they represent good indices of the fitness level and internal load (Bazzano et al., 2016; Arfuso et al., 2021b). As a matter of fact, cardiovascular evaluation is commonly performed in the equine athlete for assessment and monitoring of health, welfare and fitness. As part of this evaluation, electrocardiograms (ECG) are considered the gold standard for the diagnosis of arrhythmias in resting and exercising horses. ECG systems suffer, however, limited availability in the field, in particular during exercise, and require practitioners’ experience and knowledge. ECGs permit to visualize ventricular depolarizations and thus to measure interbeat intervals, which are required for the calculation of heart rates and heart rate variability. Heart rate is one of the most used parameters in monitoring the effects of exercise and training in horses (Vermeulen and Evans, 2006).

Knowing the internal load that is experienced by the horses during show jumping competitions is essential to develop effective training programs and to monitor whether a horse is positively adapting to the applied training. The internal load imposed on equine athletes competing in different equestrian disciplines has been thoroughly investigated in the last decades. The findings from numerous studies have considerably increased the knowledge of the internal load induced by exercise performed during eventing (Marlin et al., 1995; White et al., 1995a,b,c; Serrano et al., 2002; Kirsch et al., 2020) and endurance competitions (Rose et al., 1983; Serteyn et al., 2010; Larsson et al., 2013; Ertelt et al., 2021). However, there are still relatively few studies that evaluate the internal load that is induced by show jumping competitions (Art et al., 1990a,b; Lekeux et al., 1991; Barrey and Valette, 1993; Bazzano et al., 2016). The training and management of show jumping horses is therefore still largely based on traditional beliefs instead of scientific evidence. The relatively low speed and short duration of exercise during show jumping frequently leads to the misconception that aerobic fitness is not particularly important in this discipline. Training sessions that are specifically designed to improve aerobic capacity are therefore mostly not a regular component in conventional training programs of show jumpers (Lönnell et al., 2014). However, it has been shown that show jumping competitions elicit considerable increases in heart rate and blood lactate concentration (Art et al., 1990a,b; Lekeux et al., 1991; Barrey and Valette, 1993; Bazzano et al., 2016).

Furthermore, the jumping performance of a horse is much more difficult to assess than mere speed or endurance. Standardized field exercise test protocols usually employed to assess the physical fitness in Standardbred trotters (Couroucé, 1999; Couroucé et al., 2002) and Thoroughbred racehorses horses (Davie and Evans, 2000; Gramkow and Evans, 2006; Vermeulen and Evans, 2006) may thus be inadequate for evaluating the physical and technical capabilities required in show jumping. Consequently, the practical implementation of standardized field exercise testing for performance evaluation in show jumping horses is still very rare. Traditionally, jumping ability is evaluated based on qualitative parameters that are not only highly subjective but also require extensive experience and equestrian knowledge. Development of objective performance indicators that reflect those qualitative criteria for show jumping horses would allow to more objectively assess the horses’ performance and to monitor their long-term adaptation to training. This is especially important as show jumpers often compete year-round and perform a high number of competitions per year with minimal time for recovery between shows (Murray, 2014). Between October 2018 and September 2019, the horses ranking among the top 10 show jumping horses (according to the world ranking of the International Equestrian Federation) were participating in 12–19 (on average 16) shows and competing in 34–50 (on average 42) competitions with a mean interval of 16–26 (on average 20) days between shows (International Equestrian Federation, 2021). Regular monitoring of objective fitness indicators by means of specific exercise testing protocols for show jumping horses that can be easily integrated into the daily training routine may help to recognize whether a horse is coping with the applied workloads or not and to minimize exercise-associated injuries.

This study therefore aimed to investigate the informative value of non-specific and discipline-specific field exercise tests for performance evaluation in show jumping horses based on a retrospective analysis of data obtained from a group of horses competing at Junior and Young Rider level in a typical competition and training setting.

## Materials and Methods

### Study Design

This study was carried out as retrospective analysis of data collected as part of the “performance monitoring program” of the *German Olympic Committee for Equestrian Sports* the aim of which is to promote long-term health and performance of equine athletes by providing performance diagnostic measures. All horses to be monitored within this program were selected by the German national coach and were competing in show jumping competitions at Junior and Young Rider level.

### Show Jumping Competitions

As part of the monitoring during show jumping competitions, blood samples were collected immediately after four show jumping competitions at Junior and Young Rider level that were part of the national team selection for the European Junior and Young Rider Championship (140–150 cm) and took place during the same two events in 2019 and 2021. A total of 49 horses (24 mares, 2 stallions, and 23 geldings) aged between 8 and 17 years were sampled. Each horse was measured after one to five competitions (on average 2.0 ± 0.9 observations per horse) leading to a total of 100 observations.

### Standardized Exercise Tests

Within the scope of a 3-day training camp for Juniors and Young Riders at the German federal training center in Warendorf that was held in October 2020, a total of 12 show jumping horses of different age and levels of experience were monitored. The horses were assigned to three different performance groups based on their level of training. All horses were regularly trained and competing in show jumping competitions from national to international 3* level. Group A consisted of four horses (aged 5–7 years) with 1–2 years of competition experience, Group B consisted of four horses (aged 7–10 years) with 3–4 years of competition experience and Group C consisted of three horses (aged 10–14 years) with more than 4 years of competition experience and one horse (aged 7 years) that had less competition experience but was assigned to group C due to its high level of training. The horses were ridden throughout the whole training camp by the same four riders of the German national Junior or Young Rider squad. The same group of horses performed three different kinds of standardized exercise tests each on three consecutive days.

#### Standardized Incremental Field Exercise Test

On the first day of the 3-day training camp, all 12 horses performed a standardized incremental field exercise test (SET_track_). The horses were warmed up for approximately 10 min at walk and approximately 4 min at trot on each hand. After the warm-up, the horses performed a five-step incremental field exercise test. Each stage consisted of two laps around a 620 m sand gallop track. The aimed speed of the gallop intervals was 300, 350, 400, 450, and 500 m/min, respectively. The riders were equipped with GPS watches (Garmin Forerunner 735XT, Garmin, Olathe, KS, United States) displaying the real-time speed in order to allow them to achieve and maintain the required speed throughout the successive stages of the SET_track_. Each gallop interval was separated by a period of 30 s rest. This time period was chosen to allow blood samples to be collected between intervals. After stages 2–5, blood samples were collected immediately after finishing the gallop interval.

For practical reasons, the horses always started and finished each gallop interval at the same place and the distance of each stage was kept the same which entails that the duration of the stages was getting shorter as the speed increased.

#### Standardized Show Jumping Course

On the second day, the same group of horses performed a standardized show jumping course (SET_course_) on a 70 m × 90 m sand arena. After a warm-up phase of 10 min walk, 10 min trot, 5 min canter, and eight warm-up jumps (four uprights, four oxers), the horses performed a standardized show jumping course. The length of the course and the height and width of the fences was adjusted to the performance level of each performance group. Horses assigned to group A performed a course of 430 m length with 10 obstacles and 13 fences (one double and one triple combination) of 125–130 cm height. Horses in group B and C performed a course of 640 m length with 13 obstacles and 16 fences (one double and one triple combination). For the horses in group B the height of the fences was adjusted to 140–145 cm and for the horses assigned to group C to 145–150 cm. Blood samples were collected once immediately after finishing the course and a second time 3 min later. Video footage from two different angles was recorded throughout the SET_course_.

#### Standardized Grid Exercise

On the third day, the horses performed a standardized gridwork exercise (SET_grid_) on a 70 m x 90 m sand arena. After a warm-up phase of 10 min walk, 10 min trot, 5 min canter, and eight warm-up jumps (four uprights, four oxers), the horses performed four rounds over a grid with five oxers with one canter stride in between each fence. The heights of the fences were increased each round with the highest height performed three times in a row without rest. The height and width of the fences in the last three rounds was adjusted to the performance level of each performance group. The heights/widths of the five oxers for horses in group A were 90/90, 105/100, 115/120, 125/130, and 135/150 cm, in group B were 100/90, 115/105, 125/120, 135/135, and 145/150 cm, and in group C were 100/100, 115/115, 130/125, 140/130, and 155/150 cm. Blood samples were collected once immediately after finishing the last grid exercise and a second time 3 min later.

### Blood Sampling and Determination of Blood Lactate Concentration

Venous blood samples were collected by venipuncture of the jugular vein using 20 gauge needles and 3 mL plastic syringes. Blood lactate concentration was determined immediately after blood sample collection from whole blood by means of a hand-held photometer (Lactate Photometer Plus DP 110, Diaglobal, Berlin, Germany).

### Measurement of Heart Rate and GPS Data

During all three exercise tests, the horses were equipped with a GPS device and heart rate monitor (Equimetre Vet, Arioneo, Paris, France) which recorded the speed and heart rate throughout the whole duration of the tests at a frequency of 1 Hz. The Equimetre device consists of a sensor with two electrodes attached to a girth with one placed behind the left elbow and the other placed behind the left side of the withers under the saddle. To optimize contact, the horses’ coat and skin were wetted with water. The Equimetre system has been shown to provide accurate heart rate results compared to the Televet device in exercising horses (ter Woort et al., 2020).

### Data Processing

The data recorded during the SET_track_ were used to calculate several performance indices. Mean values of heart rate and velocity were calculated for each step of the SET_track_. The mean heart rate was calculated by averaging the heart rate values measured during each gallop interval. The mean velocity was calculated from the total distance covered during each step and the duration of each step. The resulting mean heart rate and velocity values were used to approximate the relationship between heart rate (HR) and velocity (V) by linear regression using the following formula: *HR* = *a*_1_*V* + *a*_0_. The velocities at a heart rate of 140 and 170 beats per min (V_140_ and V_170_) were calculated based on the resulting regression equations for each horse. The mean velocity and the blood lactate concentration measured after step 2–5 were used to approximate the relationship between blood lactate concentration (LAC) and velocity (V) by exponential regression using following formula: *LAC* = *a*_1_*exp*(*a*_2_*V*) + *a*_0_. The velocities at a blood lactate concentration of 2 and 4 mmol/L (V_*La2*_ and V_*La4*_) were calculated based on the individual regression equations. V_*La4*_ was extrapolated if the maximum blood lactate concentration reached during the SET_track_ was at least 3 mmol/L.

Based on the video footage recorded during SET_course_, the horses’ jumping technique and indications of muscular fatigue were rated by two independent experienced observers. In order to objectify the rating, a set of certain criteria for jumping technique (coordination, balance, responsiveness, elasticity, and mobility) and indications of muscular fatigue (loss of coordination and balance, delay in movement, slump-down during landing, longer ground contact times, frequent changes in gallop lead, loss of motivation, and decreased responsiveness) was assessed on a scale from 0 (very poor jumping technique/very clear indications for fatigue) to 10 (optimal jumping technique/no indications for fatigue at all). The grades from both observers were than averaged resulting in a total grade for each horse. For each jumping fault during SET_course_, 0.5 penalty points were subtracted from the total score. The criteria for jumping technique were rated following the evaluation guidelines for competitions for young show jumping horses specified in the German national competition regulations German Equestrian Federation (2020).

The performance score was calculated for each horse from the sum of the national ranking points achieved during the previous competition season (October–September) (German Equestrian Federation, 2021).

### Statistical Analyses

All statistical analyses were performed using R (R Core Team, 2021). In order to evaluate whether the level of difficulty, the horses’ age, the penalty score and the performance score had an effect on blood lactate concentrations in response to show jumping competitions, linear mixed effects models with level, age, penalty score, and performance score as fixed effects and horse as random effect were fitted to the data using the “lmer” function (Bates et al., 2015). Full models including all fixed effects were reduced by sequentially dropping fixed effects based on Akaike’s information criterion (AIC) which was used to assess relative model fit. Only predictors that improved the AIC by more than 2 units (ΔAIC > 2) were retained in the model (Burnham and Anderson, 2002; Arnold, 2010). The significance of the fixed effects was evaluated by comparing full models to models without the respective fixed effect by means of likelihood ratio tests using the “ANOVA” function (Bates et al., 2015). *Post hoc* pairwise comparisons between classes were done using the “emmeans” package (Lenth, 2021). Correlation between performance indices obtained from the different SETs and scores for jumping technique and resistance to fatigue was evaluated by calculating Pearson’s correlation coefficients (r) considering only pairwise complete observations using the “cor.test” function. Normality of the data was checked by visual inspection of the Q–Q (quantile–quantile) plots.

## Results

### Show Jumping Competitions

The blood lactate concentration in response to competitions at different level is shown in Table 1.

**TABLE 1:** Estimated marginal means, minima, maxima, and inter- and intra-individual coefficients of variation for blood lactate concentration in response to show-jumping competitions at different level.

	Lactate (mmol/L)						
Level (cm)	emmean (95%CI)	Min	Max	CVinter (%)	CVintra (%)	*n*	*N*
140	4.1 (3.5–4.6)^α^	1.1	8.2	39.4	35.3	28	21
145	4.7 (4.3–5.1)	1.3	7.1	30.5	20.3	51	42
150	5.2 (4.6–5.8)^β^	2.4	8.9	29.6		21	21

*emmean (95%CI), estimated marginal mean and its 95% confidence interval; CVinter (%), inter-individual coefficient of variation; CVintra (%), intra-individual coefficient of variation; n, number of observations; N, number of sampled horses.*

*Different superscripts indicate significant differences between levels.*

The level of the competition (*P* < 0.001) as well as the horses’ age (*P* = 0.001) had a significant effect on post-exercise blood lactate concentration with older horses exhibiting higher post-exercise blood lactate concentrations than younger horses. Pairwise comparisons between classes revealed a significant increase in blood lactate concentration between 140 and 150 cm classes (*P* = 0.02) but not between 140 and 145 cm (*P* = 0.14) and between 145 and 150 cm classes (*P* = 0.25). There was a significant increase in post-exercise blood lactate concentration with increasing penalty scores for horses competing in 140 cm classes (*P* = 0.02) but not for horses competing in 145 cm (*P* = 0.22) and 150 cm classes (*P* = 0.31). The horses’ performance scores did not significantly affect post-exercise blood lactate concentration (*P* = 0.69). The overall (independent of level) inter-individual coefficient of variation indicating variability of post-exercise blood lactate concentrations across horses was 31.8% and the overall intra-individual coefficient of variation indicating variability within horses was 21.4%.

### Standardized Field Exercise Tests

#### Standardized Incremental Field Exercise Test

**FIGURE 1:**
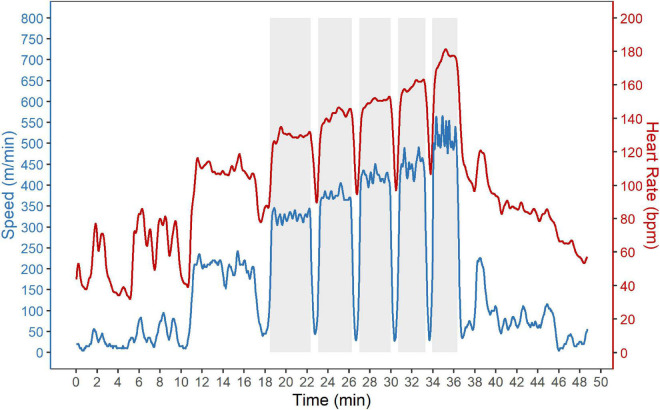
Standardized incremental field exercise test (SET_track_). Speed (blue) and heart rate (red) during the standardized 5-step incremental field exercise test on a gallop track (SET_track_) are shown exemplary for one horse. The SET_track_ consisted of a warm-up phase followed by five gallop intervals each including two laps around a 620 m sand gallop track. The aimed speed of the gallop intervals was 300, 350, 400, 450, and 500 m/min, respectively. The gallop intervals are highlighted by a gray background.

The speed and heart rate during SET_track_ are shown exemplary for one horse in Figure 1.

The mean speed and duration, mean and maximum heart rate, and blood lactate concentrations in response to the successive steps of the SET_track_ are shown in Table 2.

**TABLE 2:** Speed, heart rate and blood lactate concentration during a five-step incremental field exercise test (SET_track_).

Interval	Mean V (m/min)	Mean duration (min)	Mean HR (bpm)	Max HR (bpm)	LAC (mmol/L)
Gallop 1	314 ± 19	3.89 ± 0.19	134 ± 11	143 ± 11	
Gallop 2	357 ± 23	3.45 ± 0.2	143 ± 11	150 ± 11	0.85 ± 0.28
Gallop 3	396 ± 30	3.14 ± 0.2	150 ± 11	158 ± 14	1.26 ± 0.53
Gallop 4	438 ± 30	2.86 ± 0.19	158 ± 10	165 ± 11	1.87 ± 0.67
Gallop 5	503 ± 42	2.54 ± 0.2	171 ± 12	180 ± 13	3.57 ± 1.49

*mean V (m/min), mean speed; mean HR (bpm), mean heart rate; max HR (bpm), maximum heart rate; LAC (mmol/L), blood lactate concentration.*

The performance parameters V_140_, V_170_, V_*La2*_, and V_*La4*_ calculated from SET_track_ are shown in Table 3.

**TABLE 3:** Performance parameters derived from the standardized incremental field exercise test on a gallop track (SET_track_).

ID	Group	V_140_ (m/min)	V_170_ (m/min)	V_*La2*_ (m/min)	V_*La4*_ (m/min)
2	A	300	430	438	*
6	A	379	516	432	*
9	A	421	630	520	*
12	A	387	522	484	558
3	B	234	426	419	495
4	B	316	532	414	494
5	B	382	534	474	542
7	B	351	491	426	512
1	C	347	513	493	568
8	C	262	429	445	511
10	C	364	501	446	507
11	C	339	496	430	512

*V_140_ (m/min), velocity at a heart rate of 140 bpm; V_170_ (m/min), velocity at a heart rate of 170 bpm; V_La2_ (m/min), velocity at a blood lactate concentration of 2 mmol/L; V_La4_ (m/min), velocity at a blood lactate concentration of 4 mmol/L. V_La4_ was extrapolated if a blood lactate concentration of at least 3 mmol/L was reached in the SET_track_.*

*Not calculated (maximal lactate values < 3 mmol/L).

#### Standardized Show Jumping Course

**FIGURE 2:**
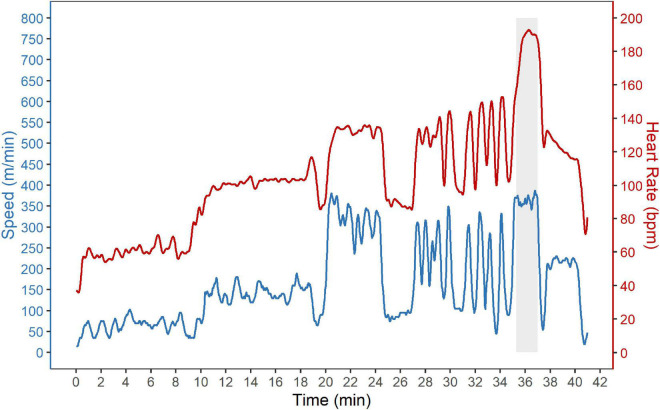
Standardized show jumping course (SET_course_). Speed (blue) and heart rate (red) during the standardized show-jumping course (SET_course_) are shown exemplary for one horse. The SET_course_ consisted of a warm-up phase followed by a standardized show-jumping course with 13 (group A) or 16 (group B and C) fences. The show-jumping course is indicated by a gray background.

The speed and heart rate during SET_course_ are shown exemplary for one horse in Figure 2.

The mean speed, distance and duration, mean and maximum heart rate, and blood lactate concentration in response to the SET_course_ are shown in Table 4.

**TABLE 4:** Scores for jumping technique and resistance to fatigue, speed, distance, duration, mean and maximum heart rate, and post-exercise blood lactate concentration in response to the standardized show-jumping course (SET_course_).

ID	Group	S_*J*_	S_*F*_	mean V (m/min)	Distance (m)	Duration (s)	Mean HR (bpm)	Max HR (bpm)	LAC_1_ (mmol/L)	LAC_3_ (mmol/L)
2	A	*	*	269	221	49	180	187	*	*
6	A	*	*	310	286	55	167	190	*	*
9	A	7.9	8.0	375	462	74	155	170	1.59	0.78
12	A	7.2	7.1	391	473	73	177	190	2.12	0.87
3	B	6.9	6.5	391	680	104	204	220	5.00	4.05
4	B	7.2	7.2	387	638	99	192	201	3.78	2.37
5	B	7.1	7.5	386	653	101	183	191	3.34	2.07
7	B	7.2	7.2	379	666	106	185	193	2.85	1.58
1	C	7.2	7.2	389	701	108	176	184	2.75	1.56
8	C	8.4	8.4	372	632	102	179	189	2.58	1.25
10	C	7.6	7.6	374	632	101	169	178	2.57	1.50
11	C	8.3	7.8	381	639	100	181	190	3.32	2.91

*S_J_, Score for jumping technique; S_F_, Score for resistance to fatigue; mean V (m/min), mean speed; mean HR (bpm), mean heart rate; max HR (bpm), maximum heart rate; LAC_1_ (mmol/L), blood lactate concentration measured immediately post-exercise; LAC_3_ (mmol/L), blood lactate concentration measured 3 min post-exercise.*

*Horses did not complete the SET_course_ due to repeated refusals, Scores were therefore not assigned and lactate was not measured.

Two horses did not finish the SET_course_ due to repeated refusals. Post-exercise blood lactate concentration was therefore not measured and scores for jumping technique and resistance to fatigue were not recorded.

#### Standardized Grid Exercise

**FIGURE 3:**
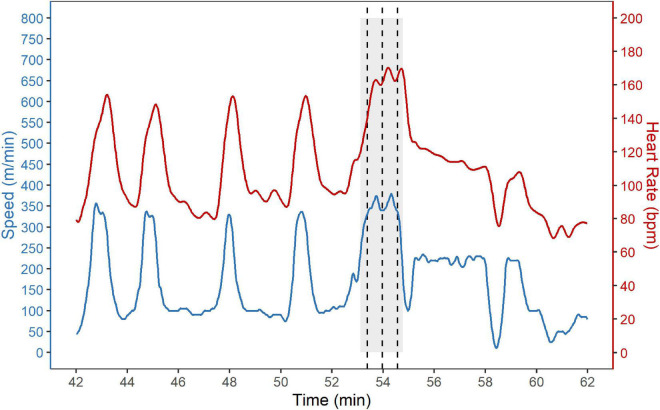
Standardized grid exercise (SET_grid_). Speed (blue) and heart rate (red) during the standardized grid exercise (SET_grid_) are shown exemplary for one horse. The SET_grid_ consisted of a warm-up phase followed by four rounds over a grid with five oxers with one canter stride in between each fence. The heights of the fences were increased each round with the highest height performed three times in a row without rest. The warm-up phase is not shown. The last three rounds through the grid are indicated by a gray background and the first jump of the grid is indicated by a dashed vertical line.

The speed and heart rate during SET_grid_ are shown exemplary for one horse in Figure 3.

The mean speed, distance and duration, mean and maximum heart rate, and blood lactate concentration in response to the SET_grid_ are shown in Table 5.

**TABLE 5:** Speed, distance, duration, mean and maximum heart rate, and post-exercise blood lactate concentration in response to the standardized grid exercise (SET_grid_).

ID	Group	Mean V (m/min)	Distance (m)	Duration (s)	Mean HR (bpm)	Max HR (bpm)	LAC_1_ (mmol/L)	LAC_3_ (mmol/L)
6	A	321	530	99	161	190	1.62	0.82
9	A	311	508	98	143	163	0.83	0.64
12	A	359	602	100	157	170	1.31	0.86
3	B	342	598	105	183	198	3.24	2.15
4	B	277	688	149	171	191	2.62	1.33
7	B	346	568	98	164	189	1.68	1.01
1	C	322	381	71	156	172	3.43	1.96
8	C	360	783	130	157	183	1.37	0.86
10	C	361	599	100	158	175	2.50	1.23
11	C	324	556	103	156	172	2.43	1.86

*mean V (m/min), mean speed; mean HR (bpm), mean heart rate; max HR (bpm), maximum heart rate; LAC_1_ (mmol/L), blood lactate concentration measured immediately post-exercise; LAC_3_ (mmol/L), blood lactate concentration measured 3 min post-exercise.*

### Correlation Between Performance Parameters

**FIGURE 4:**
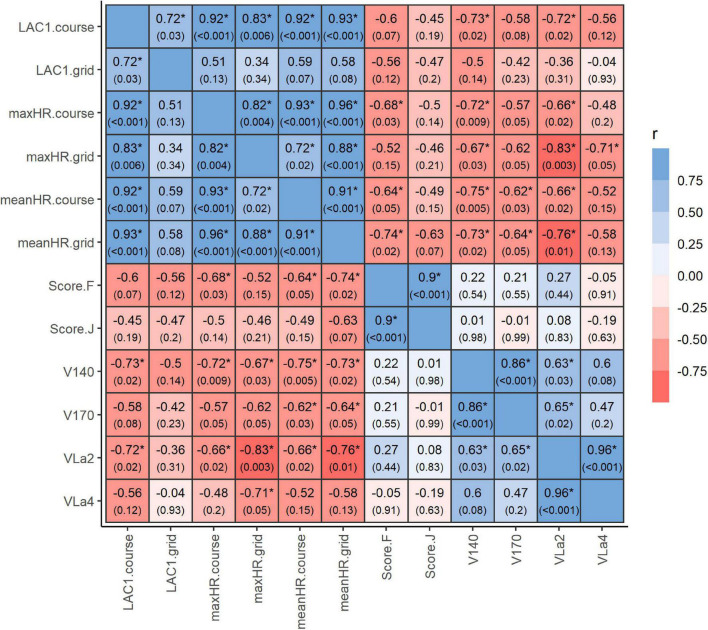
Correlation between performance parameters. Correlation between performance parameters derived from the standardized incremental field test (SET_track_) and heart rate and blood lactate in response to the standardized show-jumping course (SET_course_) and grid exercise (SET_grid_). The background color of each panel indicates the strength of the correlation between two variables (red indicates negative, blue indicates positive correlation). The correlation coefficients and *P*-values (in parenthesis) are also shown for each pair of variables. Asterisks indicate significant correlations. LAC1.course, blood lactate concentration immediately after SET_course_; LAC1.grid, blood lactate concentration immediately after SET_grid_; maxHR.course, maximum heart rate during SET_course_; maxHR.grid, maximum heart rate during SET_grid_, meanHR.course, mean heart rate during SET_course_; meanHR.grid, mean heart rate during SET_grid_; Score.F, Score for resistance to fatigue; Score.J, Score for jumping technique; V140, velocity at a heart rate of 140 bpm; V170, velocity at a heart rate of 170 bpm; VLa2, velocity at a blood lactate concentration of 2 mmol/L; VLa4, velocity at a blood lactate concentration of 4 mmol/L; r, pearson correlation coefficient.

The correlations between the different performance parameters and the score given for jumping technique and resistance to fatigue are shown in Figure 4.

There was a very strong positive correlation across mean and maximum heart rates in response to the SET_course_ and SET_grid_. The blood lactate concentrations after SET_course_ and SET_grid_, however, were strongly correlated to each other but only the lactate concentration after SET_course_ was significantly correlated to the respective heart rate values. There was a strong negative correlation of both, V_140_ and V_*La2*_ to mean and maximum heart rates during SET_course_ and SET_grid_ as well as to lactate concentration in response to SET_course_, whereas V_170_ was only significantly correlated to mean heart rate during SET_course_ and SET_grid_ and V_*La4*_ only to maximum heart rate during SET_grid_. V_140_, V_170_, and V_*La2*_ were all strongly correlated to each other but V_*La4*_ was only significantly correlated to V_*La2*_. There was no significant correlation between any of the performance indices and the score given for jumping technique but there was a strong negative correlation between the score reflecting the resistance to fatigue and the mean and maximum heart rate during SET_course_ as well as the mean heart rate during SET_grid_.

## Discussion

### Relevance of Anaerobic Capacity in Show Jumping Horses

The blood lactate concentrations measured in response to show jumping competitions at Junior and Young Rider level in the present study indicated a significant contribution of anaerobic glycolysis to energy supply. Moreover, the blood lactate concentrations in response to show jumping competitions increased with increasing level of difficulty. This effect was statistically significant even though the horses observed in this study were all competing at a relatively similar level of difficulty (140–150 cm). An increase in fence height by only 10 cm (with a concomitant increase in required speed by 50 m/min) was apparently sufficient to induce significantly higher blood lactate concentrations. These findings are consistent with previous observations in show jumping horses competing at similar level (Art et al., 1990a,b; Lekeux et al., 1991; Barrey and Valette, 1993; Bazzano et al., 2016). Various studies have shown, that although the speed required during show jumping competitions is relatively low (up to 400 m/min) and the duration of exercise is very short (up to about 90 s), completing a show jumping course induces marked changes in blood lactate concentration indicating that anaerobic glycolysis significantly contributes to energy supply during jumping exercise (Art et al., 1990a; Lekeux et al., 1991; Barrey and Valette, 1993; Aguilera-Tejero et al., 2000; Bazzano et al., 2016). In comparison to heart rates and lactate values observed in horses galloping on the flat without jumping, horses completing a show jumping course while galloping at the same speed exhibit considerably higher heart rates and blood lactate concentrations (Art et al., 1990a; Lekeux et al., 1991; van Oldruitenborgh-Oosterbaan et al., 2006). Moreover, heart rate and blood lactate concentrations during show jumping are not as strongly correlated to speed as in other equestrian disciplines such as racing or eventing (Bazzano et al., 2016). These observations indicate that the anaerobic demand is related to the additional external load that is introduced by the high metabolic requirements of muscular contraction during jumping (van Oldruitenborgh-Oosterbaan et al., 2006). The explosive power that is generated during take-off further suggests that a higher proportion of type IIX muscle fibers is recruited which primarily rely on anaerobic metabolism (Art et al., 1990a). It has therefore previously been suggested that training programs of show jumping horses should be designed to improve both aerobic and anaerobic capacity (Art et al., 1990a; Lekeux et al., 1991; Barrey and Valette, 1993). However, it is still not entirely clear as to whether the blood lactate concentrations observed in response to show jumping competitions should be interpreted as indication for the relevance of the glycolytic metabolism for jumping performance and what this implies for the design of appropriate training programs. The negative correlation between indices of aerobic capacity and blood lactate concentrations after jumping exercise as well as the huge inter-individual variability in blood lactate concentrations in response to the same level of competition observed in the present and previous studies suggest that the relevance of the anaerobic glycolysis to energy supply during show jumping depends on the horses’ aerobic capacity and jumping economy.

Feringer et al. (2019) have found that in horses with genetically higher jumping ability (European Warmbloods), the lactate transport capacity of red blood cell membranes [assessed on the basis of the expression of monocarboxylate transporter 1 (MCT1) and ancillary protein CD147] is significantly lower than in horses with lower jumping ability but higher amount of Thoroughbred blood (Brasilian Sport Horses). An increased ability to transport lactate from the blood into the red blood cells creates a concentration gradient between intramuscular and plasma lactate concentration which enhances the lactate clearance from the muscles (Koho et al., 2002). In Thoroughbred race horses that are able to gallop at very high speeds which requires a great contribution of anaerobic glycolysis to energy supply, this mechanism is believed to prevent muscle fatigue and thereby to increase racing performance (Koho et al., 2006). The low lactate transport capacity of red blood cell membranes observed in horses selected for high jumping performance on the other hand suggests that in high performance show jumpers, energy supply during jumping exercise is primarily aerobic as otherwise these horses would be prone to rapid fatigue due to acidosis (Feringer et al., 2019). Indeed, compared to blood lactate concentrations observed in Thoroughbred race horses or eventers, blood lactate concentrations in response to show jumping are considerably lower. Furthermore, it has been suggested that the low contribution of anaerobic glycolysis in horses with high-jumping ability is associated with a higher jumping economy which is probably related to a more efficient jumping technique (Feringer et al., 2019). It has been shown that there are kinematic differences in horses with high jumping ability (higher acceleration peaks in hind limbs during take-off, lower contact times, etc.) when compared to horses with lower jumping ability (Fercher, 2017; St George et al., 2021). These findings suggest that even if show jumping has an anaerobic component, the primary objective of the cardiovascular conditioning of show jumping horses should be to increase aerobic capacity in order to decrease the contribution of anaerobic glycolysis and the concomitant lactate accumulation to a minimum without reducing explosive power.

However, it should be taken into account that post-exercise blood lactate concentration does not reflect the contribution of the anaerobic alactic system which probably contributes to energy supply during jumping exercise. In humans, athletic activities that require short, all-out bursts of maximal power are considered to be highly dependent on the capacity of the alactic anaerobic system (Baker et al., 2010). More research is needed in order to better understand the relevance of the different metabolic pathways for jumping performance. One difficulty in this respect is that in contrast to the aerobic capacity which can be described by fitness indices derived from submaximal exercise tests, the anaerobic power is not as easy to determine. In human endurance athletes, the maximal lactate accumulation rate was found to be a valid parameter to describe the power of the glycolytic metabolism (Heck et al., 2003). However, such indices are usually derived from all-out sprint exercise tests which would be difficult to implement into a routine performance monitoring for show jumping horses. Discipline-specific field tests for the assessment of the capacity of the anaerobic energy metabolism in equine athletes have yet to be developed.

### Relevance of Aerobic Fitness in Show Jumping Horses

A significant relation between performance parameters indicative for aerobic capacity and competition performance has been observed in Standardbred trotters (Davie et al., 2002), Thoroughbred racehorses (Gramkow and Evans, 2006) and eventing horses (Kirsch et al., 2019). For show jumpers, however, there is no clear evidence for a direct relationship between aerobic fitness and jumping performance. Soares et al. (2016) observed that while horses competing at higher level exhibited significantly higher V_140_ values, V_180_, V_*La2*_, V_*La3*_, and V_*La4*_ were not different between performance groups. Also in the present study, there was no significant correlation between V_140_, V_170_, V_*La2*_, or V_*La4*_ and jumping performance and a significant association between penalty scores and post-exercise lactate values in response to show jumping competitions was only present in horses competing at 140 cm level competitions. This lack of evidence for a direct link between aerobic capacity and performance in show jumpers illustrates the complex nature of this discipline in which performance is not only dependent on physical ability but also skill and motivation. Nevertheless, the relevance of aerobic fitness for show jumpers should not be underestimated. It has been shown in human athletes that a high aerobic capacity enhances recovery and the resistance to fatigue during intense intermittent exercise (Tomlin and Wenger, 2001). A high aerobic capacity as indicated by a high maximum oxygen consumption decreases the contribution of anaerobic metabolism to energy supply during exercise, improves lactate removal and accelerates restoration of intramuscular energy substrates (such as creatine phosphate and glycogen). The more complete these restorative processes during recovery, the greater the ability to generate force or maintain power on subsequent work intervals (Tomlin and Wenger, 2001). In human athletes, muscular fatigue is considered to play a critical role in the development of musculoskeletal injuries as it has been shown to impair neuromuscular coordination (Carpenter et al., 1998), postural stability (Johnston et al., 1998) and control of limb velocity and acceleration (Jarić et al., 1997). In show jumpers, Assenza et al. (2016) observed higher increases in serum muscle-derived enzymes when horses were competing at a show jumping competition (two courses with 140 and 145 cm high fences on two consecutive days) that was preceded by another competition (same level) indicating that even a recovery period of 5 days did not allow for a complete muscle recovery. For show jumping horses that have to perform several bouts of high-intensity exercise within a short period of time at the same day (competitions with two rounds and/or jump-off) and on consecutive days (multi-day events), the resistance to fatigue and ability to recover quickly is therefore of paramount importance.

In the present study, lower blood lactate concentrations after jumping exercise indicating a lower proportion of anaerobic glycolysis either due to a higher aerobic fitness or a higher jumping economy or both were significantly correlated to a higher resistance to fatigue. This finding is in accordance with the results of a study by Munk et al. (2013) in which conditioning show jumping horses not only led to an increase in V_*La4*_ but was also reflected in a reduced level of fatigue during jumping exercise as perceived by the riders. These findings indicate that even if the jumping ability in itself which depends on the capacity to generate explosive power might not directly benefit from a high aerobic fitness, the ability to jump one or several complete courses without fatiguing likely does.

Another interesting finding of the present study was that older horses exhibited significantly higher blood lactate concentrations in response to the same competition level than younger horses. Léguillette et al. (2020) observed higher lactate values in response to a standardized track exercise in more experienced horses and suggested that this observation may be explained by a shift toward a higher proportion of anaerobic muscle fibers in horses trained for higher levels of show jumping competitions. However, in the present study, age did not necessarily coincide with the current level of performance. One might argue that older horses competing in Junior and Young Rider classes are often horses that did already compete at higher levels with more experienced riders and serve their purpose as “schoolmasters” for inexperienced riders. These highly experienced horses are often considered to require less training. However, these restrictive training regimens may entail a loss in physical fitness that is reflected by higher lactate values in response to showjumping competitions. Even though horses with low physical fitness but excellent jumping technique and experience may achieve relatively high levels of performance (Soares et al., 2016), those horses may be also more prone to injuries, especially if their excellent jumping technique allows them to compete at a relatively high level despite their lack of fitness. In eventing horses it has been shown that horses with a higher aerobic capacity were less likely to get injured during preparation for the European Championship than horses with lower aerobic capacity (Munsters et al., 2013).

### Comparison of Non-specific and Discipline-specific Exercise Tests in Show Jumping Horses

The physical fitness of athletic horses is usually assessed by different incremental or single-step field or treadmill exercise test protocols (Evans, 2007). On the treadmill, environmental conditions as well as speed and duration of exercise can be highly controlled, whereas field tests are more difficult to standardize but better reproduce the real environment in which horses usually train and compete (van Sloet Oldruitenborgh-Oosterbaan and Clayton, 1999; Hinchcliff et al., 2008). Field exercise tests typically consist of several incremental bouts of exercise. Based on the relationship between velocity, heart rate and blood lactate concentration during such tests, different fitness indices such as the V_*La4*_ which is considered to be a good predictor of aerobic capacity can be calculated (Couroucé-Malblanc and van Erck-Westergren, 2014). Most research on field exercise testing has focused on Standardbred trotters (Couroucé, 1999; Couroucé et al., 2002) and Thoroughbred racehorses (Davie and Evans, 2000; Gramkow and Evans, 2006; Vermeulen and Evans, 2006), but only few studies have investigated the usefulness of field exercise tests for horses competing in the Olympic equestrian disciplines (Munsters et al., 2014; Léguillette et al., 2020). Moreover, exercise tests evaluating physiological response to a prescribed speed and duration of exercise, as typically used in race horses, may be inadequate for evaluating the physical and technical capabilities required in show jumping. Léguillette et al. (2020) found that show jumpers would have to gallop at speeds of approximately 8 m/s (480 m/min) in order to experience the same internal load as during jumping over a course with 110 cm high fences. In the present study, only half of the horses reached blood lactate concentrations in excess of 4 mmol/L galloping at 500 m/min for approximately 2.5 min during the last step of SET_track_. This implies that especially horses at high levels of training would probably need to gallop at speeds around 550–600 m/min in order to reach lactate values that enable reliable determination of the onset of blood lactate accumulation and appropriately reflect the internal load induced by show jumping competitions. This however, reduces practicability of such tests as speeds considerably above 500 m/min would most likely be perceived as unusually high for a show jumping horse and the majority of riders would probably not be willing to exercise their horses at such high speeds. Moreover, the feasibility of exercise tests that require galloping at high speeds depends on the availability of an appropriate track that is sufficiently large and has a high-quality surface which is not usually available in the typical training environment of show jumping horses.

Only very few studies investigated exercise tests specifically designed for show jumpers (Munk et al., 2013; Soares et al., 2016). Munk et al. (2013) showed that a non-specific incremental exercise test as well as two kinds of discipline-specific tests, one standardized show-jumping course and one test including several rounds over 16 in-and-out jumps (maximal height of 85 cm) were suitable to monitor the horses’ response to different kinds of training programs. Soares et al. (2016) showed that heart rate and blood lactate concentration in response to a specific exercise test including jumps were more appropriate to distinguish between horses assigned to different performance groups than heart rate and lactate in response to a non-specific exercise test.

In the present study, a strong correlation between performance indices derived from non-specific and discipline-specific exercise tests was observed. However, the performance indices, obtained from the discipline-specific exercise tests SET_course_ and SET_grid_ were more strongly correlated with the scores reflecting jumping technique and resistance to fatigue than the performance indices obtained from the non-specific SET_track_. Exercise tests including jumping efforts therefore may provide additional useful information regarding jumping performance in show jumpers. The performance parameters traditionally calculated from heart rate and blood lactate concentration in response to incremental field exercise tests such as V_140_, V_170_, V_*La2*_, and V_*La4*_ primarily reflect the aerobic fitness, whereas heart rates and blood lactate concentrations in response to exercise tests including jumping efforts not only depend on aerobic fitness but also jumping economy. Although in general, horses with a higher aerobic capacity exhibited lower heart rates and blood lactate concentrations in response to jumping exercise, horses with a low aerobic capacity but a highly efficient jumping technique might still exhibit lower heart rates and blood lactate concentrations than horses with a high aerobic capacity but an inefficient jumping technique. The findings of this study therefore suggest that a comprehensive performance monitoring of show jumping horses should include both, non-specific and discipline-specific exercise tests in order to distinguish whether a horse might have deficits in aerobic fitness or jumping economy or both and to adapt the training accordingly.

Even though blood lactate concentrations measured after show jumping competitions were not directly comparable to those measured in response to SET_course_ since they were obtained from different groups of horses, it appears that the blood lactate concentrations in response to a show-jumping course in a training situation were lower than those observed after an actual competition. It has already been shown that the horses’ stress response to exercise is not only determined by the physical demands but also by their emotional state which is likely to be different in a competition situation than in a training situation (von Lewinski et al., 2013). Previous research on horses competing in an official jumping competition showed that although the highest heart rate values were recorded during and immediately after the jumping course, a rise in heart rate was already registered during the inbound to the show jumping arena indicating that the rise in heart rate is not only caused by an increase in physical activity but also reflects heightened emotional reactivity (Bazzano et al., 2016; Arfuso et al., 2021b). This suggests that a standardized course that is performed in a training situation might not reflect the internal load that is induced by a competition even if it is performed at the same level of difficulty. Monitoring heart rate and blood lactate concentrations in response to actual show jumping competitions might therefore be a valuable complement to a comprehensive performance assessment in show jumping horses.

### Limitations and Future Directions

As the horses performed the exercise tests on three consecutive days, it should be taken into account that there may have been a cumulative effect of the external load that resulted in an alteration of the internal load which is reflected in the heart rate and blood lactate concentration. It may therefore be advisable to include a recovery phase of several days between each test, however, this was not possible in the practical setting in which the data analyzed within this study have been obtained and may often not be practicable. The exercise tests were designed in accordance with the specifications of the responsible national coach with external loads reflecting those usually performed during similar training camps or competitions and the demands were adjusted to the respective level of training. Thereby it was ensured that the horse were able to cope with the applied loads without experiencing excessive fatigue.

Interestingly, the blood lactate concentration after SET_grid_ was considerably less correlated to the other performance parameters than the heart rate during SET_grid_, indicating that post-exercise blood lactate concentration might not appropriately reflect the effort of the grid exercise, probably due to the intermittent nature of this exercise. The partial recovery between each of the three rounds may have prevented lactate from accumulating in the blood. This is also reflected by the lower lactate concentrations in response to SET_grid_ than to SET_course_ despite the similar duration of exercise and number and height of jumps. A limitation of the design of the SET_grid_ in this respect was that the time interval between each of the successive rounds was not sufficiently standardized. The riders chose on their own whether they were taking a longer or shorter route which resulted in different time intervals between each round. Not only the grid itself but also the route to take and the speed to maintain between each round over the grid should therefore be standardized as well in order to increase comparability. Another limitation of this study was that jumping technique and resistance to fatigue were subjectively assessed by two independent equestrian experts on the basis of pre-defined criteria. This kind of qualitative evaluation is traditionally used to evaluate performance in show jumpers. It can be standardized to a certain degree by specifying well-defined criteria on the basis of which jumping technique and occurrence of fatigue are to be evaluated but it is still subjective and relies on the experience of the equestrian experts. Kinematic and kinetic analysis of jumping efforts may provide more objective criteria for evaluating jumping technique in horses. A study on the association between equestrian-derived performance indicators that were objectively measured by kinematic analysis and muscle activation patterns by St George et al. (2021) revealed that criteria related to impulsion and engagement such as the ability to push-off during take-off were highly correlated with the ability to generate explosive muscle power and jumping performance. However, there is still limited information on how kinematic parameters could be practically applied to routine performance evaluation in show jumping horses as they still require sophisticated equipment and complex analyses. Kinematic parameters, that can be easily measured in a practical field setting, e.g., by accelerometers may, however, have the potential to provide more objective criteria for performance monitoring in show jumpers and to improve the understanding of how physical capacity and jumping technique are related to performance (Barrey and Galloux, 1997; Fercher et al., 2016; Fercher, 2017).

## Conclusion

Both, non-specific and discipline-specific field exercise tests yielded valuable information on the individual performance capacity of show jumping horses. However, the discipline-specific tests including jumping exercise were more strongly related to jumping performance as subjectively assessed on the basis of qualitative criteria. Furthermore, discipline-specific tests are very easy to integrate into the daily training routine of show jumping horses without subjecting the horses to unusual forms of exercise which may help to increase the practicability and acceptance of regular exercise testing in show jumpers. The association between a lower contribution of anaerobic glycolysis to energy supply during jumping exercise and a greater resistance to fatigue emphasizes the importance of aerobic fitness for performance in show jumping horses. However, the role of jumping technique as well as the contribution of the alactic anaerobic metabolism to jumping performance have to be further investigated.

## Data Availability Statement

The raw data supporting the conclusions of this article will be made available by the authors, without undue reservation.

## Ethics Statement

Ethical review and approval was not required for the animal study because all data retrospectively analyzed within this study were collected for performance diagnostic purposes according to veterinary indication as part of regular competitions and training. Written informed consent was obtained from the owners for the participation of their animals in this study.

## Author Contributions

KK contributed to the study design, data collection, data processing and statistical analyses, creating figures, and writing of the manuscript. CF, SH, CR, JA, and HL contributed to the study design, data collection, and editing of the manuscript. All authors read and approved the final manuscript.

## Conflict of Interest

The authors declare that the research was conducted in the absence of any commercial or financial relationships that could be construed as a potential conflict of interest.

## Publisher’s Note

All claims expressed in this article are solely those of the authors and do not necessarily represent those of their affiliated organizations, or those of the publisher, the editors and the reviewers. Any product that may be evaluated in this article, or claim that may be made by its manufacturer, is not guaranteed or endorsed by the publisher.
